# Effects of Yeast Mannan Which Promotes Beneficial *Bacteroides* on the Intestinal Environment and Skin Condition: A Randomized, Double-Blind, Placebo-Controlled Study

**DOI:** 10.3390/nu12123673

**Published:** 2020-11-28

**Authors:** Reiko Tanihiro, Katsuhisa Sakano, Shunsuke Oba, Chikako Nakamura, Kohji Ohki, Tatsuhiko Hirota, Hiroshi Sugiyama, Shukuko Ebihara, Yasunori Nakamura

**Affiliations:** 1 Core Technology Laboratories, Asahi Quality and Innovations, Ltd., 1-21, Midori 1-chome, Moriya-shi, Ibaraki 302-0106, Japan; katsuhisa.sakano@asahi-qi.co.jp (K.S.); shunsuke.oba@asahi-qi.co.jp (S.O.); chikako.nakamura@asahi-qi.co.jp (C.N.); koji.oki@asahi-qi.co.jp (K.O.); tatsuhiko.hirota@asahi-qi.co.jp (T.H.); hiroshi.sugiyama@asahi-qi.co.jp (H.S.); yasunori.nakamura@asahi-qi.co.jp (Y.N.); 2 Chiyoda Paramedical Care Clinic, 3-3-5 Uchikanda, Chiyoda-ku, Tokyo 101-0047, Japan; info@cpcc.co.jp

**Keywords:** yeast mannan, *Bacteroides thetaiotaomicron*, *Bacteroides ovatus*, prebiotics, gut microbiota, equol, *p-*cresol, indole, skin health

## Abstract

Yeast mannan (YM) is an indigestible water-soluble polysaccharide of the yeast cell wall. In vitro fecal fermentation studies showed that YM could exhibit a notable prebiotic effect. The aim of this randomized, double-blind, placebo-controlled study was to assess the efficacy of YM intake on the intestinal environment and skin condition. One hundred and ten healthy female subjects aged 30–49 years were supplemented with YM or placebo for eight weeks. Skin dryness was set as the primary endpoint. No side effects were observed during the study. Microbiota analyses revealed that YM intake selectively increased the relative abundance of *Bacteroides thetaiotaomicron* and *Bacteroides ovatus* compared to that by placebo. Feces and urine analyses showed that YM intake lowered the concentration of fecal *p*-cresol, indole, and skatole, and elevated urinal equol levels compared to those in placebo. Furthermore, YM supplementation ameliorated subjective skin dryness. This study suggests that YM intake could promote beneficial *Bacteroides* and improve the intestinal environment and skin condition.

## 1. Introduction

Yeast has long been consumed as an ingredient in a large variety of fermented foods and beverages such as bread, beer, and wine. Yeast mannan (YM) is a water-soluble, low-viscosity, indigestible polysaccharide derived from the yeast cell wall and has rarely been used as a food ingredient. It is composed of a highly branched mannose polymer with a molecular weight of 20–200 kDa [1]. Furthermore, it includes a linear α-1,6-mannoside backbone branched with α-1,2-mannoside and α-1,3-mannoside bonds in mono-, di-, tri-, and tetramer forms [1,2]. This structure is distinct from other plant cell wall mannans, such as konjac glucomannan and carob galactomannan, which include only β-linked mannose and not α-linked mannose [3].

In our previous study, we reported that YM was utilized by fecal microbiota and selectively increased the relative abundance of *Bacteroides thetaiotaomicron* and *Bacteoroides ovatus* in two types of in vitro assay systems [4,5]. This is explained by the distinctive gene apparatuses in these two *Bacteroides* species: mannan-specific polysaccharide utilization loci contributed to α-mannan partial degradation into oligosaccharides, membrane transport of oligosaccharides, and further degradation of oligosaccharides [6]. *B. thetaiotaomicron* and *B. ovatus*, which are the dominant species in the human colon [7], have been previously shown to be safe [8,9] and are expected to be utilized as potential novel probiotics. *B. thetaiotaomicron* was reported to perform numerous functions, including the promotion of anti-rotavirus activity [10], induction of matrilysin [11], and attenuation of bowel inflammation [12]. Furthermore, the *B. thetaiotaomicron* has been used as a live biotherapeutic compound for Crohn’s disease [8]. *B. ovatus* is known for its capacity to express the tumor-specific Thomsen-Friedenreich antigen [13] and alleviate lipopolysaccharide-induced inflammation [14]. Furthermore, *B. ovatus* is known as an equol-producing bacterium [15]. Equol, which is produced by gut microbes from daidzein, exerts higher estrogenic activity than daidzein, a major type of soy isoflavone [15]. Equol has been reported to attenuate menopausal symptoms [16], premenstrual syndrome [17], and skin aging [18]. Our previous study using an in vitro assay showed that YM suppressed indole and phenol production [4]. Furthermore, it indicated that mannan was the main component responsible for the YM’s capacity to suppress the indole and phenol production. Phenolic compounds (phenols), including phenol, *p*-cresol, and 4-ethylphenol, as well as indolic compounds (indoles), such as indole and skatole, are putrefactive metabolites produced by gut bacteria from dietary tyrosine and tryptophan respectively [19], and are closely related to skin condition [20]. It remains unclear whether YM exerts prebiotic effects in vivo. We hypothesized that YM intake might increase the beneficial *Bacteroides* abundance, suppress putrefactive compound levels, and improve skin condition in humans. In this study, a randomized, double-blind, placebo-controlled trial was conducted to explore this hypothesis and evaluate beneficial effects on the gut and skin.

## 2. Materials and Methods

### 2.1. Study Design

This clinical trial was approved by the Institutional Review Board of Chiyoda Paramedical Care Clinic (Approval Number 19071904, Tokyo, Japan) and was carried out in accordance with the ethical principles of the Declaration of Helsinki and the ethical guidelines for medical and health research involving human subjects. This study was registered with the University Hospital Medical Information Network (UMIN) Clinical Trial Registry as UMIN000037734 and was conducted in compliance with the protocol. Written informed consent was obtained from all subjects prior to enrolment. The study was designed as a double-blind, randomized, placebo-controlled, parallel clinical trial. The experimental design is summarized in Table 1. Subjects were assigned to two groups using blocked randomization based on their ages, skin hydration, and transepidermal water loss (TEWL) values. Allocation to the YM or placebo group was concealed from subjects, investigators, technicians, data analysts, evaluators, and the medical doctor, until after the experiment was complete.

Subjects were referred to the Chiyoda Paramedical Care Clinic (Tokyo, Japan) five times (the pretrial test, 0, 4, and 8 weeks after administration, as well as at the end of the examination). Daily records from two weeks prior the start of treatment (–2 weeks) until the end of the examination were kept. The records included information regarding treatment intake, the presence of menstruation, physical condition (the presence of any symptoms), intake of other drugs, and a defecation log. During the pretrial test, the inner forearm skin hydration and TEWL were evaluated. For the urinal analysis, before the two-week period preceding the treatment start, all subjects were instructed to take a soy isoflavone supplement once daily for three days. The urine samples were collected after three days of soy isoflavone supplementation. Before the start of the treatment (0 weeks), fecal samples were collected, constipation assessment was performed (using the constipation assessment scale, CAS), skin measurements were performed, a skin condition questionnaire was completed, and a medical interview was performed for each subject. These tests were repeated throughout the study, as indicated in Table 1. The subjects were instructed to take five test tablets (YM or placebo) once daily for eight weeks. After the 8th week measurement, all subjects were instructed to supplement with soy isoflavone once daily for three days. Urine samples were collected the day following the last day of supplementation with soy isoflavone. Subjects were instructed to continue ingesting test tablets (YM or placebo) once daily over the second urinal sampling. Skin dryness was set as a primary endpoint. The study was performed from August 2019 to January 2020.

### 2.2. Subjects

Healthy female volunteers between 30 and 49 years of age with a stable menstrual period (between 28–35 days) and aware of their skin dryness were recruited to participate in this trial. We selected 110 subjects with inner forearm TEWL levels and skin hydration values close to the averages of the total recruited participants. The number of subjects was calculated from preliminary study results for healthy female volunteers. Briefly, subjects with the same conditions as this study were grouped together. The relative abundance of *B. thetaiotaomicron* in the YM and placebo groups after 8 weeks of intake were compared. The mean difference was 0.717%, and the standard deviation (SD) was 1.29. We used G* Power 3.1.9.2 software to calculate the required sample size assuming α = 0.05 and β = 0.20 (80% power) [21]. The resulting sample size was 52 subjects per group. Therefore, assuming a 5% dropout rate, the target number of subjects was set at 110 (55 subjects per group).

The exclusion criteria were as follows: (1) serving yoghurt more than three times per week, (2) serving a beverage containing lactic acid bacteria or bifidobacteria more than twice per week, (3) habitual use of drug or medicine that might affect the results of the study, (4) undergoing antibiotic treatment within a month, (5) reoccurring constipation and diarrhea, (6) self-reporting defecation frequency as lower than 4 times/week or higher than 8 times/week, (7) pregnancy, lactation, or planning to become pregnant, (8) risk of food allergy, (9) participation in any other clinical trials within a month, (10) planning to go traveling abroad during this study, and (11) other reason for ineligibility as judged by the principal investigator. The subjects were instructed to maintain their previous lifestyle habits and avoid long-time trips and long-term outdoor activities throughout the trial period. We excluded from the efficacy analysis those (1) who took less than 85% of the expected number of tablets, (2) who did not keep an adequate log or whose behavior cast doubt on the reliability of their clinical data, (3) who met the exclusion criteria after enrollment or who did not follow the study guidelines, and (4) for whom there were justifiable reasons for exclusion.

### 2.3. Test Tablets

YM was produced from yeast cell wall slurry provided by Asahi Group Foods, Ltd. (Tokyo, Japan) as previously described [4]. A previous study indicated that the suppressed production of fecal putrefactive compounds associated with YM supplementation was mainly attributed to mannan content [4]. Subjects in the YM group were instructed to ingest five active tablets, containing 1.1 g of YM once daily. It contained 0.62 g of mannan based on a measurement method described in a previous report [5]. The placebo tablets were prepared using the same procedures and formula except for YM, which was replaced with maltose. The tablets contained crystalline cellulose, calcium stearate, and silicon dioxide.

### 2.4. Fecal Microbiota Analysis

Fecal samples were collected at 0, 4, and 8 weeks, and fecal DNA was extracted using the bead beating method previously described [22]. The OneStep PCR Inhibitor Removal Kit (Zymo Research, Tustin, CA, USA) was used for further purification according to the manufacturer’s instructions. Purified DNA was stored at –20 °C until use. Quantitative polymerase chain reaction

(qPCR) analyses were performed using an Applied Biosystems QuantStudio 3 Real-Time PCR system (Thermo Fisher Scientific Inc., Waltham, MA, USA). The qPCR experiments were carried out using specific primers for all eubacteria and five *Bacteroides* species (*B. thetaiotaomicron*, *B. ovatus*, *B. vulgatus, B. uniformis*, and *B. fragilis*), as previously described [23,24]. The qPCR amplification program is described in Supplementary Table S1. All eubacteria were detected using the SsoAdvanced Universal SYBR Green Supermix (Bio-Rad Laboratories, Hercules, CA, USA) and the five *Bacteroides* species were detected using the Premix Ex Taq™ (Probe qPCR) (Takara Bio, Shiga, Japan). We prepared a synthesized DNA fragment (207–290 bp) identical to the 16S rRNA gene sequence which was used as a reference for absolute quantification of each method.

Next-generation sequencing of the bacterial V4 region encoding the 16S rRNA gene was performed using a MiSeq system (Illumina Inc., San Diego, CA, USA) and MiSeq Reagent Kit version 2 (300 Cycle). The sequencing was carried out according to a previous report using 12.5 ng of DNA from each fecal sample [25]. These sequence data have been submitted to the DNA Data Bank of Japan (DDBJ) Sequence Read Archive under accession number DRA011139. QIIME ver.1.8.0 was used to filter and analyze the sequences [26]. Quality filtering was performed using the provided fastq files, and sequences with a quality score under 29 were removed. Chimeric sequences were removed using USEARCH, and operational taxonomic units (OTUs) were assigned using open-reference OTU picking with a 97% threshold of pairwise identity. After OTUs containing <5 sequences were removed, the OTUs were classified taxonomically using the Greengenes reference database [27].

### 2.5. Measurement of Fecal Samples

Indoles (indole and skatole) and phenols (*p-*cresol, phenol, and 4-ethylphenol) from feces were measured as previously described [28] using gas chromatography and mass spectrometry. The concentrations of acetic acid, propionic acid, *n*-butyric acid, isobutyric acid, *n*-valeric acid, and isovaleric acid were measured using high-performance liquid chromatography (HPLC), as previously described by Ikeda et al. [29] (Supplementary Figure S1). To measure the water content of the fecal samples, the weighted samples were heated at 80 °C for more than 16 h. After cooling, the weight of the residue was measured, and the amount of water contained in the sample was calculated. To measure the pH of the fecal samples, the samples were diluted 1:10 (*wt:wt*) with distilled water and heated at 85 °C for 15 min. The supernatant was measured with a pH meter LAQUAtwin B-712 (HORIBA scientific Ltd., Kyoto, Japan). The concentration of ammonium ions in feces was measured using an ion chromatography system as previously described [28].

### 2.6. Stool Properties and Bowel Habits

All subjects recorded the number of their bowel movements and fecal characteristics every day starting two weeks prior to the beginning of treatment until the end of the examination. Fecal characteristics were assessed using the Bristol Stool Scale (BSS) [30] recorded for each defecation. The constipation status was assessed using the Japanese version of the CAS composed of eight items [31,32]. A high score is indicative of severe constipation. The subjects filled out the CAS form at 0, 4, and 8 weeks based on their condition one-week prior to the visits.

### 2.7. Urinal Equol

To provide a precursor of equol, soy isoflavone tablets (25 mg) were used. Soy isoflavone tablets, produced by Asahi Group Foods Ltd., contained reduced palatinose, soy germ extracts, calcium silicate, calcium stearate, and crystalline cellulose. The first urine of the morning was collected on the day following the last intake of soy isoflavone tablet. Intake of soybeans, soybean-processed foods, and foods containing soy isoflavone were prohibited on the day before urine sampling. Equol and its precursors, daidzein and dihydrodaidzein, were identified and quantified using an ultra high performance liquid chromatography (UHPLC) procedure [33] with modifications. Based on the method described in the previous study [33], we modified composition of the mobile phases to use ammonium formate instead of phosphate. In our study, one of the mobile phases was water with 5 mM ammonium formate and the other was methanol with 5 mM ammonium formate.

### 2.8. Skin Condition

The subjective skin condition was assessed using a Likert scale questionnaire [34] regarding skin dryness and skin itchiness. The subjects filled out the questionnaire at the 0 and 8th week visits based on their condition one week prior to the visits. Subjects evaluated their skin condition using a 5-point system ranging from 1 (good condition) to 5 (bad condition). A high score was indicative of severe skin condition. During the pretrial test, the inner forearm skin parameters were measured, while during the visits at 0 and 8 weeks, the cheeks and inner forearm skin parameters were measured. To evaluate skin hydration and TEWL levels, Corneometer CM825 and Tewameter TM300 (Courage and Khazaka Electronic GmbH, Cologne, Germany) were used, respectively. All measurements of skin condition were performed in supinated position in a testing room with stable temperature (21 ± 1 °C) and humidity (50% ± 5%).

### 2.9. Safety Evaluation

Safety was evaluated using medical interviews, diary contents, physical examinations, and blood examinations. The occurrence of adverse events (AEs) and side effects were monitored in subjects who consumed test tablets at least once. The medical interview and physical examinations (body weight, systolic and diastolic blood pressure, and pulse rate) were performed during each visit. Body height was measured only during the pretrial test. The body mass index (BMI) at each time point was calculated using the body height data obtained in the pretrial test. Blood samples were collected during the pretrial test and the 8th week visit. General biochemical examination of the blood samples was performed by the LSI Medience Corporation (Tokyo, Japan).

### 2.10. Statistical Analysis

All statistical analyses were performed using the IBM SPSS Statistics 20 (IBM Japan Ltd., Tokyo, Japan) software. Parametric and non-parametric tests were performed using unpaired samples Student’s *t*-test and Mann–Whitney U test, respectively (for parameters measured twice throughout the study). Repeated measures two-way analysis of variance (ANOVA) was used to analyze the parameters measured three times throughout the study. Incidence rates of AEs were compared using the chi-squared test. For the defecation frequency analysis, the average value per week in each period described below was used: (1) from –2 weeks to the start of treatment, (2) from 3 weeks to 4 weeks, and (3) from 7 weeks to 8 weeks. For the Bristol stool scale (BSS) analysis, the average score in each period described above was used. Differences were considered significant for *p*-values < 0.05.

## 3. Results

### 3.1. Characteristics of the Subjects

Two hundred and ninety-four subjects were recruited from Tokyo, Japan. Following the study protocol, 110 subjects (healthy females) were enrolled. The subjects were randomly assigned to the YM group (n = 55) or the placebo group (n = 55). Two subjects from the YM group dropped out during the intervention period due to personal reasons not related to this study. A total of 108 subjects aged 30 to 49 years completed this study. Eight subjects were excluded from the efficacy analysis due to the following reasons: one subject from the YM group violated the restrictions, one subject from the YM group and one from the placebo group were excluded due to the use of antibiotics, one subject from the YM group and two from the placebo group were judged inappropriate based on the diary log of excess defecation frequency during the pre-intake period, and one subject from the YM group and one from the placebo group were excluded due to the lack of menstruation throughout the study period. Finally, the data for 100 subjects (49 in the YM group and 51 in the placebo group) were used for the efficacy analysis. Three subjects from the YM group and one from the placebo group were excluded from the gut microbiota composition analysis using next-generation sequencing because during the next-generation sequencing, the polymerase chain reaction for the V4 region of the bacterial 16S rRNA encoding gene did not yield sufficient amplicons. One subject from the placebo group was excluded from the urinal analysis since none of the three metabolites, daidzein, dihydrodaidzein, and equol, were detected in the urine sample. One subject from the YM group was excluded from the defecation frequency and BSS score analyses due to the ingestion of a laxative for more than three days. One subject from the YM group experienced a fever eight days before the skin measurement, thus, she was judged inadequate for the objective skin parameter analysis. The inner forearm TEWL data of two subjects from the YM group were excluded due to their abnormal values (less than 1.0 g/m^2^h). These abnormal values might have been caused by errors in the measurement procedure. Thus, the efficacy was analyzed using Per protocol set. The AEs and side effect safety analyses included 110 subjects (55 subjects per group), while the safety analysis set for the laboratory test included 108 subjects (53 in the YM group and 55 in the placebo group) who completed this trial.

The background characteristics of the subjects who completed the trial are shown in Table 2. No significant differences were observed in age, BMI, systolic blood pressure, diastolic blood pressure, pulse rate, skin hydration, and TEWL during the pretrial test. The ingestion rates for the test samples were 99.3% ± 2.1% and 99.6% ± 1.2% (mean ± standard deviation (SD)) in the YM and placebo groups, respectively.

### 3.2. Fecal Microbiota

**Figure 1:**
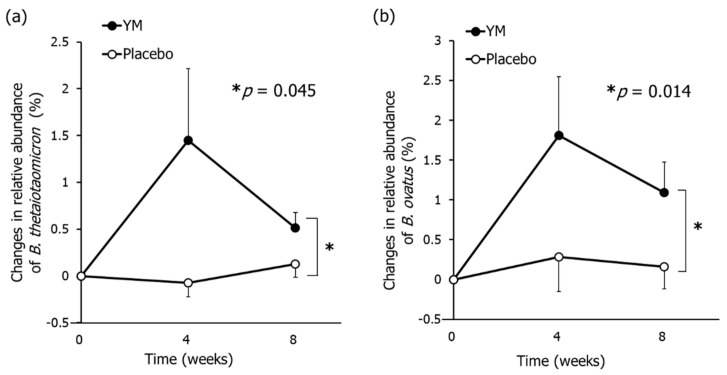
Effects of YM intake on the relative abundance of *B. thetaiotaomicron* and *B. ovatus* in feces. Changes in relative abundance of *B. thetaiotaomicron* (**a**) and *B. ovatus* (**b**). The black circles represent the YM group and the white circles represent the placebo group. Data represent the means ± standard error (SE). The *p*-values were measured using repeated-measures analysis of variance (ANOVA) for the differences between groups without multiple comparison correction. Asterisks indicate overall significance between groups across time points (* *p* < 0.05).

The prebiotic effects of YM were evaluated using qPCR and the amount of changes in relative abundance of five *Bacteroides* species predominantly harbored in the human colon were measured [35,36]. The changes in the relative abundance of *B. thetaiotaomicron* and *B. ovatus* in the YM group were larger than those in the placebo group (Figure 1), while no significant changes in relative abundance of *B. vulgatus*, *B. uniformis*, and *B. fragilis* were observed between groups (Supplementary Table S2).

Further modifications of the bacterial communities following YM intake were examined using next-generation sequencing. The taxon abundances that averaged over 1% at baseline were used for the statistical analysis. The sole genus *Streptococcus* was found to be significantly different between groups at baseline (2.18% ± 0.66% in the YM group compared to 0.53% ± 0.17% in the placebo group). Three genera had significantly different abundance throughout the intervention between groups, although they had no statistical difference at baseline between groups. All of them, *Ruminococcus*, *Ruminococcaceae;g*, and *Clostridiales;f;g*., belong to the *Clostridiales* order (Table 3). *Ruminococcaceae;g* is an unclassified genus belonging to the *Ruminococcaceae* family, while *Clostridiales;f;g* is an unclassified genus belonging to the *Clostridiales* order. For each of these genera, the relative abundance in the YM group was lower than that in the placebo group. On the other hand, any genera abundance in the YM group were not significantly higher than those in the placebo group throughout the intervention. YM intake did not increase any genera abundance, but significantly increased the abundance of the two *Bacteroides* species.

### 3.3. Bowel Habits and Constipation Symptoms

**Figure 2:**
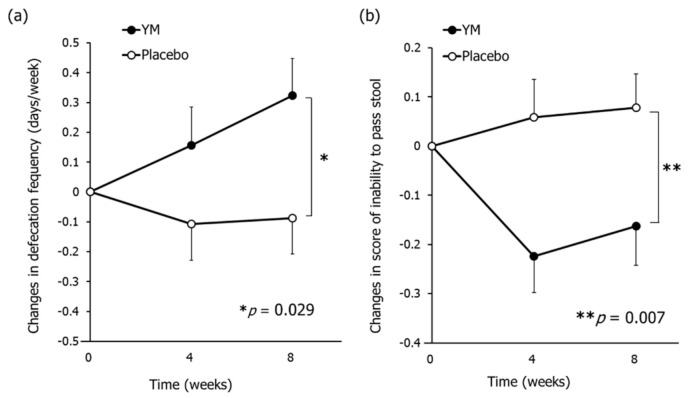
Effects of YM intake on constipation symptoms. Changes in defection frequency (days/week) (**a**) and CAS score of inability to pass stool (**b**). The black circles represent the YM group and the white circles represent the placebo group. Data represent the means ± SE. The *p*-values were measured using repeated-measures ANOVA for the differences between groups without multiple comparison correction. CAS: Constipation assessment scale. Asterisks indicate overall significance between groups across time points (* *p* < 0.05, ** *p* < 0.01).

Following treatment, the changes in defecation frequency (days/week) were significantly larger in the YM group than in the placebo group (Figure 2a), while there was no significant difference in the changes in defecation frequency (times/week) between groups. As shown in Supplementary Table S3, the defecation frequency (days/week) during the pre-intake period was 5.3 ± 0.2 in the YM group and 5.4 ± 0.2 in the placebo group. Accordingly, YM intake normalized bowel habits to approach daily defecation. The changes in fecal water content throughout the intervention were significantly larger in the YM group than those in the placebo group (Supplementary Table S3). On the other hand, there was no significant difference in BSS scores between groups. In this study, the distribution of BSS scores at the baseline were concentrated around 4 (optimal score) (3.8 ± 0.1 for both groups).

The constipation symptoms were assessed using CAS, and the results are summarized in Supplementary Table S4. YM intake significantly lowered the several scores such as “Inability to pass stool”, “Less frequent bowel movements”, and “Small volume of stool” compared to those in placebo (Figure 2b, Supplementary Table S4). There was no difference between groups in score changes for “abdominal distention or bloating”, “change in amount of gas passed rectally”, “rectal fullness or pressure”, “rectal pain with bowel movements”, “oozing liquid stool”, and total CAS score. Collectively, YM intake improved the defecation frequency, moistened feces, and alleviated certain constipation symptoms.

### 3.4. Fecal and Urinal Metabolites

**Figure 3:**
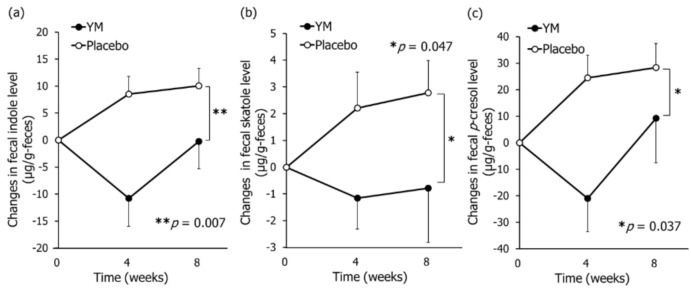
Effects of YM intake on fecal putrefactive compound levels. Changes in fecal concentrations of indole (**a**), skatole (**b**), and *p-*cresol (**c**). Black circles represent the YM group and the white circles represent the placebo group. Data represent the means ± SE. These were determined by repeated-measures ANOVA between groups without multiple comparison correction. Asterisks indicate overall significance between groups across time points (* *p* < 0.05, ** *p* < 0.01).

As shown in Figure 3, YM intake significantly lowered fecal indole, skatole, and *p-*cresol levels compared to those in placebo. Additionally, YM intake significantly lowered fecal ammonia levels and the sum of fecal phenols and indoles levels compared to those in placebo (Supplementary Table S5).

Except for the decreased isovalerate levels observed in the YM group compared to the placebo group *(p* = 0.048), no other changes in short-chain fatty acids (acetate, propionate, butyrate, isobutyrate, and valerate) were observed between groups. Although a previous study suggested that the fecal concentration of isovalerate was positively correlated with constipation symptoms [37], further research is required in order to clarify this association.

**Figure 4:**
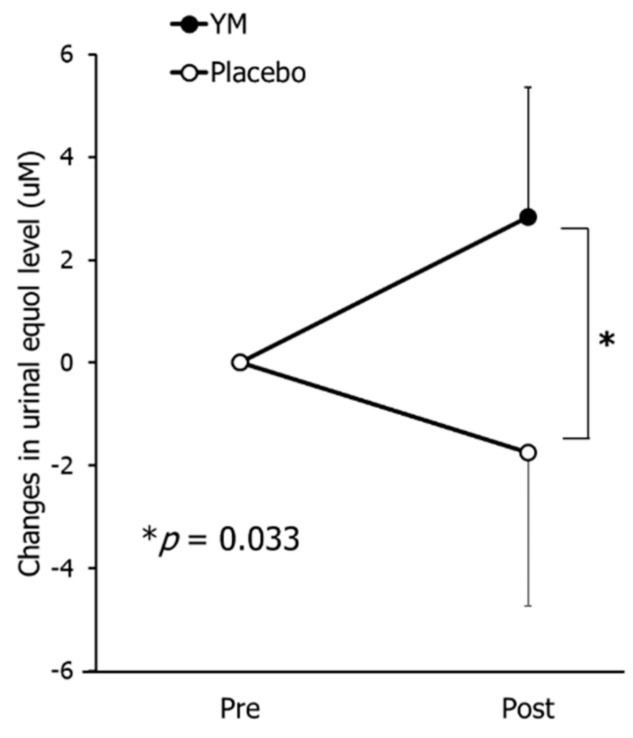
Effects of YM intake on urinal equol level. Black circles represent the YM group and the white circles represent the placebo group. Data represent the means ± SE. Data were analyzed by the Mann–Whitney U test. Asterisk indicates significant differences between groups (* *p* < 0.05).

As shown in Figure 4, following treatment, the changes in urinal equol level in the YM group were significantly higher than those in the placebo group (*p* = 0.033), confirming that YM intake elevated equol production from daidzein.

### 3.5. Skin Condition

**Figure 5:**
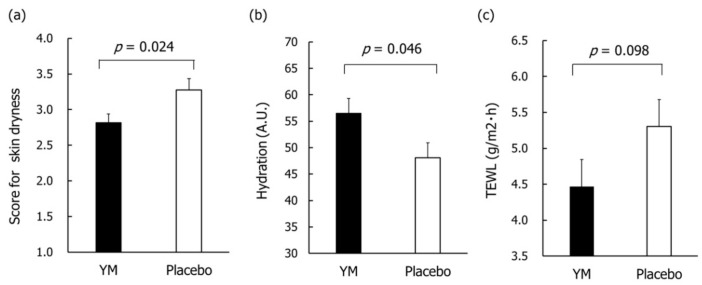
Effects of YM intake on skin conditions after 8 weeks of intervention. Data represent the means ±SE. Questionnaire of skin dryness (**a**). The *p-*value was estimated using the Mann–Whitney U test. Cheek hydration (**b**), and inner forearm TEWL (**c**) in the subjects with dry-skin type. A.U.: arbitrary unit. TEWL: transepidermal water loss. The *p-*values were calculated using unpaired samples Student’s *t*-test between YM and placebo groups.

Based on the skin condition questionnaire, there were no between group differences in both scores of skin dryness and skin itching at baseline, respectively. The scores of skin dryness after 8 weeks of intervention were significantly lower in the YM group than those in the placebo group (Figure 5a), while there was no significant difference in skin itching scores after 8 weeks of intervention between groups (*p* = 0.479).

Furthermore, the effects of YM intake on skin hydration and TEWL were evaluated. There were no between group differences in those values at baseline, while there were also no differences in those after 8 weeks of intervention between groups. A subgroup analysis that targeted subjects with higher than average baseline cheek TEWL levels was performed to assess the effects of YM intake on objective skin dryness in subjects whose skin barrier function are lower. There were no between group differences in skin hydration and TEWL levels at baseline. The cheek hydration value in the YM group was significantly higher than that in the placebo group after 8 weeks of intervention (Figure 5b). The inner forearms TEWL levels in the YM group tended to be lower than those in the placebo group after 8 weeks of intervention (Figure 5c).

### 3.6. Safety

We observed no side effects and no severe or moderate AEs throughout the intervention. During the trial, 117 mild AEs were recorded. Of these, 58 were recorded in the YM group (*n* = 26) and 59 were recorded in the placebo group (*n* = 30). The incidence rates of AEs among groups were comparable, with 47.3% (26/55) in the YM group and 54.5% (30/55) in the placebo group. Five of the most common mild AEs were cough, sore throat, runny nose, headache associated with a cold, and menstrual pain. AEs related to YM intake were not observed. Therefore, the principle investigator reported no safety issues associated with YM intake. The laboratory test results indicated that there are no clinically significant findings associated with YM intake.

## 4. Discussion

Prebiotics are defined as “selectively fermented ingredients that result in specific changes in the composition and/or activity of the gastrointestinal microbiota, thus conferring benefits upon host health” [38], and most traditional prebiotics increase abundance of bifidobacteria or lactic acid bacteria. Recent studies on intestinal bacteria revealed that bifidobacteria, lactic acid bacteria, and a variety of gut microbes play beneficial roles in the regulation of host health. Recently, the concept of beneficial microbes has been evolving. Based on the current perspective, the capability to manipulate a variety of intestinal microbes appears to be beneficial for health maintenance. Most prebiotics selectively increase the abundance of gut microbes at the genus level, but few studies have reported on prebiotics that selectively increase the abundance of gut microbes at the species level in humans. Therefore, we investigated YM as a new potential prebiotic that increases the abundance of specific beneficial bacterial species in humans.

*Bacteroides* is one of the most predominant genera of bacteria in the human colon. Among the 53 species classified into the *Bacteroides* genus, *B. thetaiotaomicron*, *B. ovatus*, *B. vulgatus, B. uniformis,* and *B. fragilis* are predominant in the human colon [35,36]. Some of them have been proposed to have harmful effects on human health. *B. fragilis* was associated with the induction of abscess formation [39], while *B. vulgatus* was associated with the development of ulcerative colitis [40]. The qPCR analysis revealed that YM intake increased the abundance of beneficial *Bacteroides* species, *B. thetaiotaomicron* and *B. ovatus*, and did not affect the abundance of harmful *Bacteroides* species in humans. This is consistent with the results of a previous in vitro study performed by our group [5]. Furthermore, the present study showed that YM intake could induce species-specific changes in the intestinal microbiota using qPCR and next-generation sequencing. The increase in *B. thetaiotaomicron* abundance might contribute to the anti-viral and anti-enteric pathogen protection and alleviate bowel inflammation due to its specific properties as a gut symbiont [10,11,12].

The defecation dairies and CAS analysis revealed that YM supplementation had health benefits which are observed in the general dietary fiber intervention, such as improvement of defecation frequency and amelioration of constipation symptoms. Additionally, we observed that YM could suppress fecal *p-*cresol, indole, and skatole levels in humans. This is partially supported by the results of an in vitro study using rat feces, where YM was used to suppress phenol levels (a precursor of *p-*cresol) [4]. The result differences might be explained by the difference in gut microbiota between humans and rats. Recently, several ingredients composed of soluble dietary fibers, such as wheat bran extract, inulin-type fructan, and hydrolyzed guar gam, have been reported to suppress either phenols or indoles [41,42,43]. Since no dietary fiber ingredient suppressed both phenols and indoles simultaneously, it was speculated that YM might suppress *p-*cresol and indoles through a different mechanism. Furthermore, there are differences between the necessary YM dose for an effective treatment, and other dietary fibers. For wheat bran extract, inulin-type fructan, and hydrolyzed guar gam, a daily dose of 10, 10, and 21 g respectively [41,42,43], was necessary to suppress phenols or indoles, while the intake of 1.1 g/day of YM induced *p-*cresol and indoles suppression. *B. thetaiotaomicron* has been shown to ameliorate colon inflammation in preclinical models of Crohn’s disease [12]. Crohn’s disease patients usually exhibit a decreased relative abundance of *Bacteroides* compared to healthy individuals, while the relative abundance of *Ruminococcus* is increased compared to healthy individuals [44,45]. *Ruminococcus* and *Ruminococcaceae* are strongly associated with the production of *p*-cresol and indoles [46,47]. YM intake might exert its suppressive effects on *p*-cresol and indoles by increasing the abundance of *B. thetaiotaomicron*, which leads to decrease the abundance of *Ruminococcus* and *Ruminococcaceae,* thus inhibiting the production of *p*-cresol and indoles. Nevertheless, further studies are required to clarify the mechanism through which YM suppresses fecal *p-*cresol and indole levels. These results suggest that YM intake could diminish the toxicological risk of tyrosine and tryptophan metabolites present in the gastrointestinal tract, resulting in an improved intestinal environment.

Our previous studies focused on the effect of YM on the intestinal environment [4,5], while the present study further highlighted the effect of YM intake on the urinal equol levels following soy isoflavone supplementation. *B. ovatus* is known as an equol-producing bacterium [15], and its relative abundance is shown to be negatively correlated with premenstrual syndrome [48]. Although some food ingredients, such as polydextrose, L-arabinose, and xylitol, have been shown to promote equol production in animal studies [49,50,51], these results cannot be directly translated to humans since equol levels in humans are lower than those in rodents [15]. To the best of our knowledge, little has been reported on food ingredients that significantly increased equol levels in human. Our findings suggest that YM might represent a new prebiotic, which can be further developed into products that might benefit women’s wellbeing [16,17,18]. The gut-skin axis represented by the association between the intestinal environment and skin health, has become highly investigated recently [52]. We suggest that the observed effect of YM intake on the skin condition might be exerted through putrefactive compounds such as *p-*cresol, since the harmful effect of such compounds on the skin are well known [20]. We have conducted this study in the subjects enrolled based on the averages of both TEWL levels and skin hydration values. In future, its beneficial effect on the objective skin dryness will be revealed by conducting the study that recruits subjects whose skin barrier function are lower than this trial. In this study, YM intake improved the feeling of dry skin and constipation such as “Inability to pass stool”, “Less frequent bowel movements”, and “Small volume of stool”. Women are particularly conscious of improving skin and constipation, therefore YM could be expected to match the needs of women.

Our study has several limitations. It has been reported that skin moisture content changes with age [53,54]. In this study, participant age range was set tight to evaluate the effect of YM intake by reducing variation between participants. Because the structure of the skin is the same depending on gender and age group, it is speculated that YM intake can be expected to be effective for a wider range of subjects. However, further studies were needed to verify the YM’s effect in males or other age groups. The intestinal microbial populations of our participants might have been affected by their diets. For instance, the putrefactive compound levels in the stool might be affected by the intake of meat, which generally contains large amounts of tyrosine and tryptophan, which are converted into putrefactive compounds by gut microbes. Although the subjects were instructed to avoid fecal sample collection after ingesting excess meat dishes, we did not impose strict dietary regulations or to supply them with uniform diets. While this study focused on the effect of YM intake on equol production, it remains unclear whether the effect of YM intake on equol levels exert beneficial physiological effects.

## 5. Conclusions

The present study suggests that YM is a safe and functional ingredient with prebiotic effects, which can increase the relative abundance of *B. thetaiotaomicron* and *B. ovatus* in humans. The daily intake of YM affected the composition of metabolites generated by gut microbiota, thus suppressing indole, skatole, and *p-*cresol production and elevating equol levels. Additionally, YM intake exerted a beneficial effect on subjective skin dryness and partially improved the objective skin dryness.

## Figures and Tables

**Table 1:** Study schedule.

Events	Pretrial Test	Period of Time (Weeks)				The End of the Examination
		–2	0	4	8	
Visit	●		●	●	●	●
Test-samples intake			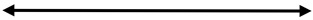			
Soy-isoflavone intake	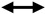				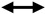	
Urine sampling		●				●
Feces sampling			●	●	●	
CAS			●	●	●	
Measurement of skin	●		●		●	
Questionnaire of skin			●		●	
Blood sampling	●				●	
Medical interview	●		●	●	●	●
Diary	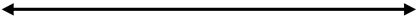					

Subjects were selected according to the pretrial test, followed by a two-week pre-intake period and an eight-week treatment period. Each period of soy isoflavone ingestion was three days. CAS: Constipation assessment scale. The arrow means to do it daily in the indicated period.

**Table 2:** Background characteristics of subjects at the pretrial test.

Parameters	Unit	YM Group	Placebo Group	*p-*Value
Age	years	40.7 ± 5.6	40.7 ± 5.7	0.995
Body mass index (BMI)	kg/m^2^	20.5 ± 2.2	21.0 ± 2.8	0.298
Systolic blood pressure	mmHg	108.7 ± 10.9	110.4 ± 12.6	0.461
Diastolic blood pressure	mmHg	66.0 ± 8.4	67.8 ± 9.6	0.310
Pulse rate	bpm	71.0 ± 8.0	73.3 ± 10.6	0.219
Hydration level (inner forearm)	-	30.3 ± 4.0	29.4 ± 4.1	0.295
TEWL (inner forearm)	g/m^2^h	6.0 ± 1.0	5.9 ± 1.1	0.905

Data represent the mean ± standard deviation (SD). The *p*-values were calculated using unpaired samples Student’s *t*-tests. TEWL: transepidermal water loss; YM: Yeast mannan.

**Table 3:** Comparison of fecal microbiota between the YM and placebo group.

Genus	Groups	Relative Abundance (%)			*p-*Value
		Baseline	4 Weeks	8 Weeks	
*Bifidobacterium*	YM	9.1 ± 1.4	6.7 ± 1.1	6.6 ± 1.0	0.062
	Placebo	6.2 ± 0.8	4.2 ± 0.6	5.1 ± 0.9	
*Bacteroides*	YM	20.1 ± 1.9	27.7 ± 2.5	26.8 ± 2.1	0.076
	Placebo	17.8 ± 1.8	21.8 ± 1.8	22.9 ± 1.9	
*Parabacteroides*	YM	1.4 ± 0.3	2.0 ± 0.5	2.3 ± 0.4	0.064
	Placebo	1.2 ± 0.2	1.3 ± 0.2	1.3 ± 0.1	
*Rikenellaceae;g*	YM	1.4 ± 0.2	1.7 ± 0.4	2.0 ± 0.4	0.392
	Placebo	2.1 ± 0.5	2.0 ± 0.3	2.2 ± 0.4	
Clostridiales;f;g *	YM	0.8 ± 0.2	0.4 ± 0.1	0.9 ± 0.3	0.018
	Placebo	1.9 ± 0.5	1.9 ± 0.5	2.5 ± 0.6	
*Lachnospiraceae;g*	YM	4.3 ± 0.6	4.7 ± 0.6	4.6 ± 0.6	0.693
	Placebo	4.7 ± 0.4	4.9 ± 0.4	4.7 ± 0.4	
*Blautia*	YM	11.8 ± 1.4	8.7 ± 1.0	8.0 ± 1.2	0.942
	Placebo	11.2 ± 1.2	8.3 ± 0.9	8.7 ± 0.9	
*Coprococcus*	YM	5.6 ± 0.8	4.6 ± 0.6	4.6 ± 0.7	0.082
	Placebo	7.2 ± 0.8	6.0 ± 0.7	5.9 ± 0.6	
*Roseburia*	YM	4.0 ± 0.8	3.0 ± 0.5	3.1 ± 0.5	0.343
	Placebo	4.3 ± 0.7	3.7 ± 0.6	3.9 ± 0.6	
*Lachnospiraceae;g[Ruminococcus]*	YM	4.0 ± 0.8	2.8 ± 0.4	2.4 ± 0.3	0.340
	Placebo	2.5 ± 0.5	2.6 ± 0.3	2.7 ± 0.4	
Ruminococcaceae;g **	YM	0.8 ± 0.2	0.7 ± 0.2	0.7 ± 0.2	0.008
	Placebo	1.6 ± 0.4	2.1 ± 0.4	1.9 ± 0.4	
*Faecalibacterium*	YM	14.5 ± 1.4	14.1 ± 1.4	14.1 ± 1.3	0.844
	Placebo	14.6 ± 1.2	15.8 ± 1.1	13.2 ± 1.0	
*Oscillospira*	YM	2.1 ± 0.3	1.9 ± 0.2	2.4 ± 0.3	0.424
	Placebo	2.2 ± 0.2	2.4 ± 0.2	2.4 ± 0.2	
Ruminococcus *	YM	2.3 ± 0.4	2.2 ± 0.3	2.5 ± 0.4	0.015
	Placebo	3.2 ± 0.4	3.4 ± 0.4	3.8 ± 0.4	

Data represent the mean ± SE. The *p*-values were calculated using repeated-measures ANOVA for the differences between groups without multiple comparison correction. Asterisks indicate overall significance between groups across time points (* *p* < 0.05, ** *p* < 0.01). Genera that showed significantly different abundance between groups at baseline were excluded.

## Supplementary Materials

The following are available online at https://www.mdpi.com/2072-6643/12/12/3673/s1: Figure S1: The typical chromatographic profiles of feces samples, Table S1: qPCR amplification conditions for each bacterial species, Table S2: Comparison of relative abundance of *Bacteroides* species between the YM and placebo group, Table S3: Comparison of bowel habits and fecal conditions between the YM and placebo group, Table S4: Comparison of CAS scores between the YM and placebo group, Table S5: Comparison of fecal putrefactive compound levels between the YM and placebo group.

## References

[1] Liu H.Z., Liu L., Hui H., Wang Q. (2015). Structural characterization and antineoplastic activity of *Saccharomyces cerevisiae* mannoprotein. Int. J. Food Prop..

[2] Kocourek J., Ballou C.E. (1969). Method for fingerprinting yeast cell wall mannans. J. Bacteriol..

[3] Scheller H.V., Ulvskov P. (2010). Hemicelluloses. Annu. Rev. Plant Biol..

[4] Oba S., Washida K., Shimada Y., Sunagawa T., Tanihiro R., Sugiyama H., Nakamura Y. (2020). Yeast mannan increases *Bacteroides thetaiotaomicron* abundance and suppresses putrefactive compound production in in vitro fecal microbiota fermentation. Biosci. Biotechnol. Biochem..

[5] Oba S., Sunagawa T., Tanihiro R., Awashima K., Sugiyama H., Odani T., Nakamura Y., Kondo A., Sasaki D., Sasaki K. (2020). Prebiotic effects of yeast mannan, which selectively promotes *Bacteroides thetaiotaomicron* and *Bacteroides ovatus* in a human colonic microbiota model. Sci. Rep..

[6] Cuskin F., Lowe E.C., Temple M.J., Zhu Y., Cameron E., Pudlo N.A., Porter N.T., Urs K., Thompson A.J., Cartmell A. (2015). Human gut Bacteroidetes can utilize yeast mannan through a selfish mechanism. Nature.

[7] Martens E.C., Lowe E.C., Chiang H., Pudlo N.A., Wu M., McNulty N., Abbott D.W., Henrissat B., Gilbert H.J., Bolam D.N. (2011). Recognition and degradation of plant cell wall polysaccharides by two human gut symbionts. PLoS Biol..

[8] A Phase I Randomized, Double-Blind, Placebo-Controlled Study to Assess the Safety and Tolerability of (Thetanix™) Bacteroides thetaiotaomicron in Adolescents with Stable Crohn’s Disease. https://www.4dpharmaplc.com/application/files/1815/5824/8886/Thetanix_DDW_poster_2019.pdf.

[9] Tan H., Yu Z., Wang C., Zhang Q., Zhao J., Zhang H., Zhai Q., Chen W. (2018). Pilot Safety Evaluation of a Novel Strain of *Bacteroides ovatus*. Front. Genet..

[10] Varyukhina S., Freitas M., Bardin S., Robillard E., Tavan E., Sapin C., Grill J., Trugnan G. (2012). Glycan-modifying bacteria-derived soluble factors from *Bacteroides thetaiotaomicron* and *Lactobacillus casei* inhibit rotavirus infection in human intestinal cells. Microbes. Infect..

[11] López-Boado Y.S., Wilson C.L., Hooper L.V., Gordon J.I., Hultgren S.J., Parks W.C. (2000). Bacterial exposure induces and activates matrilysin in mucosal epithelial cells. J. Cell Biol..

[12] Delday M., Mulder I., Logan E.T., Grant G. (2019). *Bacteroides thetaiotaomicron* Ameliorates Colon Inflammation in Preclinical Models of Crohn’s Disease. Inflamm. Bowel Dis..

[13] Ulsemer P., Henderson G., Toutounian K., Löffler A., Schmidt J., Karsten U., Blaut M., Goletz S. (2013). Specific humoral immune response to the Thomsen-Friedenreich tumor antigen (CD176) in mice after vaccination with the commensal bacterium *Bacteroides ovatus* D-6. Cancer Immunol. Immunother..

[14] Tan H., Zhao J., Zhang H., Zhai Q., Chen W. (2019). Novel strains of *Bacteroides fragilis* and *Bacteroides ovatus* alleviate the LPS-induced inflammation in mice. Appl. Microbiol. Biotechnol..

[15] Yuan J., Wang J.H., Liu X. (2007). Metabolism of dietary soy isoflavones to equol by human intestinal microflora—Implications for health. Mol. Nutr. Food Res..

[16] Ishiwata N., Melby M.K., Mizuno S., Watanabe S. (2009). New equol supplement for relieving menopausal symptoms: Randomized, placebo-controlled trial of Japanese women. Menopause.

[17] Takeda T., Shiina M., Chiba Y. (2018). Effectiveness of natural S-equol supplement for premenstrual symptoms: Protocol of a randomised, double-blind, placebo-controlled trial. BMJ Open.

[18] Oyama A., Ueno T., Uchiyama S., Aihara T., Miyake A., Kondo S., Matsunaga K. (2012). The effects of natural S-equol supplementation on skin aging in postmenopausal women: A pilot randomized placebo controlled trial. Menopause.

[19] Bakke O. (1969). Studies on the degradation of tyrosine by rat caecal contents. Scand. J. Gastroenterol..

[20] Miyazaki K., Masuoka N., Kano M., Iizuka R. (2014). *Bifidobacterium* fermented milk and galacto-oligosaccharides lead to improved skin health by decreasing phenols production by gut microbiota. Benef. Microbes.

[21] Faul F., Erdfelder E., Lang A.G., Buchner A. (2007). G*Power 3: A flexible statistical power analysis program for the social, behavioral, and biomedical sciences. Behav. Res. Methods.

[22] Hatanaka M., Yamamoto K., Suzuki N., Iio S., Takara T., Morita H., Takimoto T., Nakamura T. (2018). Effect of *Bacillus subtilis* C-3102 on loose stools in healthy volunteers. Benef. Microbes.

[23] Tong J., Liu C., Summanen P., Xu H., Finegold S. (2011). Application of quantitative real-time PCR for rapid identification of *Bacteroides fragilis* group and related organisms in human wound samples. Anaerobe.

[24] Furet J., Firmesse O., Gourmelon M., Bridonneau C., Tap J., Mondot S., Dor´e J., Corthier G. (2009). Comparative assessment of human and farm animal faecal microbiota using real-time quantitative PCR. FEMS Microbiol. Ecol..

[25] Imoto N., Morita H., Amanuma F., Maruyama H., Watanabe S., Hashiguchi N. (2018). Maternal antimicrobial use at delivery has a stronger impact than mode of delivery on bifidobacterial colonization in infants: A pilot study. J. Perinatol..

[26] Caporaso J.G., Kuczynski J., Stombaugh J., Bittinger K., Bushman F.D., Costello E.K., Fierer N., Peña A.G., Goodrich J.K., Gordon J.I. (2010). QIIME allows analysis of high-throughput community sequencing data. Nat. Methods.

[27] The Greengenes Database. http://greengenes.secondgenome.com/?prefix=downloads/greengenes_database/gg_13_5/.

[28] Aoe S., Nakamura F., Fujiwara S. (2018). Effects of wheat bran on fecal butyrate-producing bacteria and wheat bran combined with barley on *Bacteroides* abundance in Japanese healthy adults. Nutrients.

[29] Ikeda N., Saito Y., Shimizu J., Ochi A., Mizutani J., Watabe J. (1994). Variations in concentrations of bacterial metabolites, enzyme activities, moisture, pH and bacterial composition between and within individuals in faeces of seven healthy adults. J. Appl. Bacteriol..

[30] O’Donnell L.J., Virjee J., Heaton K.W. (1990). Detection of pseudodiarrhoea by simple clinical assessment of intestinal transit rate. BMJ.

[31] Nagaviroj K., Yong W.C., Fassbender K., Zhu G., Oneschuk D. (2011). Comparison of the constipation assessment scale and plain abdominal radiography in the assessment of constipation in advanced cancer patients. J. Pain Symptom Manag..

[32] Fukai K., Sugita A., Tanaka M. (1995). A developmental study of the Japanese version of the constipation assessment scale. JJNS.

[33] Redruelloa B., Guadamurob L., Cuestaa I., Álvarez-Buyllaa J.R., Mayob B., Delgado S. (2015). A novel UHPLC method for the rapid and simultaneous determination of daidzein, genistein and equol in human urine. J. Chromatogr. B.

[34] Likert R. (1932). A technique for the measurement of attitudes. Arch. Psychol..

[35] Shilnikova I.I., Dmitrieva N.V. (2015). Evaluation of antibiotic susceptibility of *Bacteroides, Prevotella and Fusobacterium* species isolated from patients of the N. N. Blokhin Cancer Research Center, Moscow, Russia. Anaerobe.

[36] Oh H., Amin N.E., Davies T., Appelbaum P.C., Edlund C. (2001). gyrA mutations associated with quinolone resistance in *Bacteroides fragilis* group strains. Antimicrob. Agents Chemother..

[37] Watanabe J. (2005). Carbonhydrate fermentation in the colon. J. Intest. Microbiol..

[38] Roberfroid M., Gibson G.R., Hoyles L., McCartney A.L., Rastall R., Rowland I., Wolvers D., Watzl B., Szajewska H., Stahl B. (2010). Prebiotic effects: Metabolic and health benefits. Br. J. Nutr..

[39] Tzianabos A.O., Onderdonk A.B., Rosner B., Cisneros R.L., Kasper D.L. (1993). Structural features of polysaccharides that induce intra-abdominal abscesses. Science.

[40] Bamba T., Matsuda H., Endo M., Fujiyama Y. (1995). The pathogenic role of *Bacteroides vulgatus* in patients with ulcerative colitis. J. Gastroenterol..

[41] François I.E.J.A., Lescroart O., Veraverbeke W.S., Marzorati M., Possemiers S., Evenepoel P., Hamer H., Houben E., Windey K., Welling G.W. (2012). Effects of a wheat bran extract containing arabinoxylan oligosaccharides on gastrointestinal health parameters in healthy adult human volunteers: A double-blind, randomised, placebo-controlled, cross-over trial. Br. J. Nutr..

[42] Li L., Xiong Q., Zhao J., Lin X., He S., Wu N., Yao Y., Liang W., Zuo X., Ying C. (2020). Inulin-type fructan intervention restricts the increase in gut microbiome-generated indole in patients with peritoneal dialysis: A randomized crossover study. Am. J. Clin. Nutr..

[43] Okubo T., Ishihara N., Takahashi H., Fujisawa T., Kim M., Yamamoto T., Mitsuoka T. (1994). Effects of partially hydrolyzed guar gum intake on human intestinal microflora and its metabolism. Biosci. Biotechnol. Biochem..

[44] Zhou Y., Zhi F. (2016). Lower level of *Bacteroides* in the gut microbiota is associated with inflammatory bowel disease: A meta-analysis. Biomed. Res. Int..

[45] Png C.W., Lindén S.W., Gilshenan K.S., Zoetendal E.G., McSweeney C.S., Sly L.I., McGuckin M.A., Florin T.H.J. (2010). Mucolytic bacteria with increased prevalence in IBD mucosa augment in vitro utilization of mucin by other bacteria. Am. J. Gastroenterol..

[46] Ji M., Du H., Xu Y. (2020). Structural and metabolic performance of *p*-cresol producing microbiota in different carbon sources. Food Res. Int..

[47] Amaretti A., Gozzoli C., Simone M., Raimondi S., Righini L., Pérez-Brocal V., Rodrigo García-López R., Moya A., Rossi M. (2019). Profiling of protein degraders in cultures of human gut microbiota. Front Microbiol..

[48] Matsumoto M., Iino H., Suganuma H. Intestinal Microbiota Correlate to Menstrual Cycle-Dependent Complaints and Plasma Levels of Female Hormones. Proceedings of the Annual Meeting of Intestinal Microbiology.

[49] Tamura M., Hori S., Nakagawa H. (2010). Impact of dietary polydextrose on the daidzein metabolism in adult mice. Biosci. Microflora.

[50] Tamura M., Kurusu Y., Hori S. (2012). Effect of dietary L-arabinose on the intestinal microbiota and metabolism of dietary daidzein in adult mice. Biosci. Microbiota Food Health.

[51] Tamura M., Hoshi C., Hori S. (2013). Xylitol affects the intestinal microbiota and metabolism of daidzein in adult male mice *Int*. J. Mol. Sci..

[52] Salem I., Ramser A., Isham N., Ghannoum M.A. (2018). The gut microbiome as a major regulator of the gut-skin axis. Front Microbiol..

[53] Leveque J.L., Corcuff P., De Rigal J., Agache P. (1984). *In vivo* studies of the evolution of physical properties of the human skin with age. Int. J. Dermatol..

[54] Darlenski R., Sassning S., Tsankov N., Fluhr J.W. (2009). Non-invasive in vivo methods for investigation of the skin barrier. Eur. J. Pharm. Biopharm..

