# Supplementary material for: Effects of Yeast Mannan Which Promotes Beneficial Bacteroides on the Intestinal Environment and Skin Condition: A Randomized, Double-Blind, Placebo-Controlled Study
Source: Nutrients. 2020 Nov 28;12(12):3673. doi: 10.3390/nu12123673 (PMC7761098; doi:10.3390/nu12123673)
Supplement: Supplementary file 1 [file nutrients-12-03673-s001.pdf]

Supplementary Information

Title

Effects of Yeast Mannan which Promotes Beneficial *Bacteroides* on the Intestinal Environment and Skin Condition: A Randomized, Double-Blind, Placebo-Controlled Study

Reiko Tanihiro [1,*], Katsuhisa Sakano [1], Shunsuke Oba [1], Chikako Nakamura [1], Kohji Ohki [1], Tatsuhiko Hirota [1], Hiroshi Sugiyama [1], Shukuko Ebihara [2], and Yasunori Nakamura [1]

[1] Core Technology Laboratories, Asahi Quality and Innovations, Ltd., 1-21, Midori 1-chome, Moriya-shi, Ibaraki 302-0106, Japan; katsuhisa.sakano@asahi-qi.co.jp (K.S.); shunsuke.oba@asahi-qi.co.jp (S.O.); chikako.nakamura@asahi-qi.co.jp (C.N.); koji.oki@asahi-qi.co.jp (K.O.); tatsuhiko.hirota@asahi-qi.co.jp (T.H.); hiroshi.sugiyama@asahi-qi.co.jp (H.S.); yasunori.nakamura@asahi-qi.co.jp (Y.N.)

[2] Chiyoda Paramedical Care Clinic; info@cpcc.co.jp

*Correspondence: reiko.tanihiro@asahi-qi.co.jp; Tel.: +81-297-46-9347

Table S1. qPCR amplification conditions for each bacterial species.

Target species	Primer-F<br>Primer-R (5' to 3')<br>Probe (5' FAM - 3' TAMRA)	Strains for standard curves	PCR amplification condition	Ref.
All eubacteria	CGGTGAATACGTTCCCGG<br>TACGGCTACCTTGTTACGACTT	*B. fragilis*<br>ATCC 25285	95 °C for 10 s,<br>[95 °C for 20 s, 56 °C for 20 s,<br>and 72 °C for 30 s] × 40 cycles	24
B. thetaiotaomicron	GCAAACTGGAGATGGCGA<br>AAGGTTTGGTGAGCCGTTA<br>TCGATGGGGATGCGTTCCATTAGG	*B. thetaiotaomicron*<br>ATCC 29741	95 °C for 30 s,<br>[95 °C for 15 s and 62.5 °C for 60 s]<br>× 40 cycles	23
B. ovatus	TGCAAACTRAAGATGGC<br>CAAACTAATGGAACGCATC<br>CACGTATCCAACCTGCCGATAACTCC	*B. ovatus*<br>ATCC 8483	95 °C for 30 s,<br>[95 °C for 15 s and 58 °C for 60 s]<br>× 40 cycles	23
B. vulgatus	CGGGCTTAAATTGCAGATGA<br>CATGCAGCACCTTCACAGAT<br>TGAAAGCCGTAAGCCGCAAGG	*B. vulgatus*<br>ATCC 8482	95 °C for 30 s,<br>[95 °C for 15 s and 63 °C for 60 s]<br>× 40 cycles	23
B. uniformis	TCTTCCGCATGGTAGAACTATTA<br>ACCGTGTCTCAGTTCCAATGTG<br>CGTTCCATTAGGTTGTTGGCGGGG	*B. uniformis*<br>ATCC 8492	95 °C for 30 s,<br>[95 °C for 15 s and 60 °C for 60 s]<br>× 40 cycles	23
B. fragilis	TCRGGAAGAAAGCTTGCT<br>CATCCTTTACCGGAATCCT<br>ACACGTATCCAACCTGCCTTTACTCG	*B. fragilis*<br>ATCC 25285	95 °C for 30 s,<br>[95 °C for 15 s and 63 °C for 60 s]<br>× 40 cycles	23

Table S2. Comparison of relative abundance of *Bacteroides* species between the YM and placebo group.

Species	Treatment	Changes from baseline					
		4 Weeks			8 Weeks		
B. thetaiotaomicron *	YM	1.45	±	0.76	0.51	±	0.17
	Placebo	-0.07	±	0.15	0.13	±	0.14
*B. ovatus**	YM	1.81	±	0.74	1.09	±	0.38
	Placebo	0.28	±	0.43	0.16	±	0.28
B. vulgatus	YM	0.59	±	0.33	0.28	±	0.35
	Placebo	0.23	±	0.23	0.23	±	0.20
B. uniformis	YM	0.40	±	0.26	0.31	±	0.36
	Placebo	-0.06	±	0.32	0.12	±	0.32
B. fragilis	YM	0.09	±	0.05	0.02	±	0.03
	Placebo	-0.06	±	0.14	-0.11	±	0.13

Data represent the mean ± standard error (SE). Asterisks indicate overall significance between groups across time points measured using repeated measures ANOVA for the changes from baseline without multiple comparison correction (* $p < 0.05$).

Table S3. Comparison of bowel habits and fecal conditions between the YM and placebo group.

Item	Unit	Treatment	Baseline			4 Weeks			8 Weeks		
Defecation days *	days/week	YM	5.3	±	0.2	5.5	±	0.2	5.6	±	0.2
		Placebo	5.4	±	0.2	5.2	±	0.2	5.3	±	0.2
Defecation times	times/week	YM	5.9	±	0.2	6.1	±	0.2	6.5	±	0.3
		Placebo	5.7	±	0.2	5.7	±	0.2	5.9	±	0.2
BSS		YM	3.8	±	0.1	3.8	±	0.1	3.9	±	0.1
		Placebo	3.8	±	0.1	3.9	±	0.1	3.8	±	0.1
Fecal water contents**	%	YM	72.4	±	1.0	74.3	±	1.0	73.4	±	0.9
		Placebo	74.7	±	0.9	72.7	±	0.9	72.4	±	0.9

Data represent the mean ± SE. BSS: Bristol Stool Scale. Asterisks indicate overall significance between groups across time points measured using repeated measures ANOVA for the changes from baseline without multiple comparison correction (* $p < 0.05$, ** $p < 0.01$).

Table S4. Comparison of CAS scores between the YM and placebo group.

Item	Treatment	Baseline	4 Weeks	8 Weeks
Inability to pass stool**	YM	0.43 ± 0.08	0.20 ± 0.07	0.27 ± 0.06
	Placebo	0.33 ± 0.07	0.39 ± 0.07	0.41 ± 0.07
Less frequent bowel movements*	YM	0.08 ± 0.04	0.08 ± 0.04	0.04 ± 0.03
	Placebo	0.08 ± 0.04	0.18 ± 0.06	0.16 ± 0.05
Small volume of stool*	YM	0.14 ± 0.05	0.06 ± 0.03	0.04 ± 0.03
	Placebo	0.04 ± 0.03	0.10 ± 0.04	0.08 ± 0.04
Abdominal distention or bloating	YM	0.31 ± 0.07	0.33 ± 0.07	0.31 ± 0.07
	Placebo	0.45 ± 0.08	0.25 ± 0.06	0.35 ± 0.07
Change in amount of gas passed rectally	YM	0.27 ± 0.08	0.33 ± 0.08	0.20 ± 0.07
	Placebo	0.18 ± 0.07	0.12 ± 0.05	0.18 ± 0.07
Rectal fullness or pressure	YM	0.39 ± 0.08	0.27 ± 0.06	0.24 ± 0.06
	Placebo	0.31 ± 0.07	0.39 ± 0.07	0.31 ± 0.07
Rectal pain with bowel movements	YM	0.10 ± 0.04	0.12 ± 0.05	0.16 ± 0.05
	Placebo	0.06 ± 0.03	0.06 ± 0.03	0.12 ± 0.05
Oozing liquid stool	YM	0.10 ± 0.04	0.10 ± 0.04	0.12 ± 0.05
	Placebo	0.16 ± 0.05	0.12 ± 0.05	0.14 ± 0.05
Total CAS score	YM	1.82 ± 0.27	1.49 ± 0.24	1.39 ± 0.20
	Placebo	1.61 ± 0.22	1.61 ± 0.23	1.75 ± 0.22

Data represent the mean ± SE. CAS: Constipation assessment scale. Asterisks indicate overall significance between groups across time points measured using repeated measures ANOVA for the changes from baseline without multiple comparison correction (* $p < 0.05$, ** $p < 0.01$).

Table S5. Comparison of fecal putrefactive compound levels between the YM and placebo group.

Fecal metabolite	Unit	Treatment	Changes from baseline					
			4 Weeks			8 Weeks		
Indole**	μg/g-feces	YM	-10.8	±	5.2	-0.3	±	5.0
		Placebo	8.5	±	3.3	10.0	±	3.2
p -Cresol*		YM	-21.0	±	12.6	9.2	±	16.8
		Placebo	24.4	±	8.6	28.3	±	9.0
Skatole*		YM	-1.2	±	1.1	-0.8	±	2.0
		Placebo	2.2	±	1.3	2.8	±	1.2
Phenol		YM	-2.1	±	1.9	-2.6	±	1.5
		Placebo	-1.9	±	1.0	-2.1	±	1.7
4-Ethylphenol		YM	-0.1	±	0.6	-0.2	±	0.5
		Placebo	0.3	±	0.5	0.2	±	0.5
Sum of phenols and indoles*		YM	-35.2	±	17.3	5.4	±	22.4
		Placebo	33.5	±	11.5	39.3	±	12.1
Ammonia*		YM	-59.4	±	49.5	60.3	±	50.4
		Placebo	61.9	±	36.4	187	±	49.2

Data represent the mean ± SE. Asterisks indicate overall significance between groups across time points measured using repeated measures ANOVA for the changes from baseline without multiple comparison correction (* $p < 0.05$, ** $p < 0.01$).

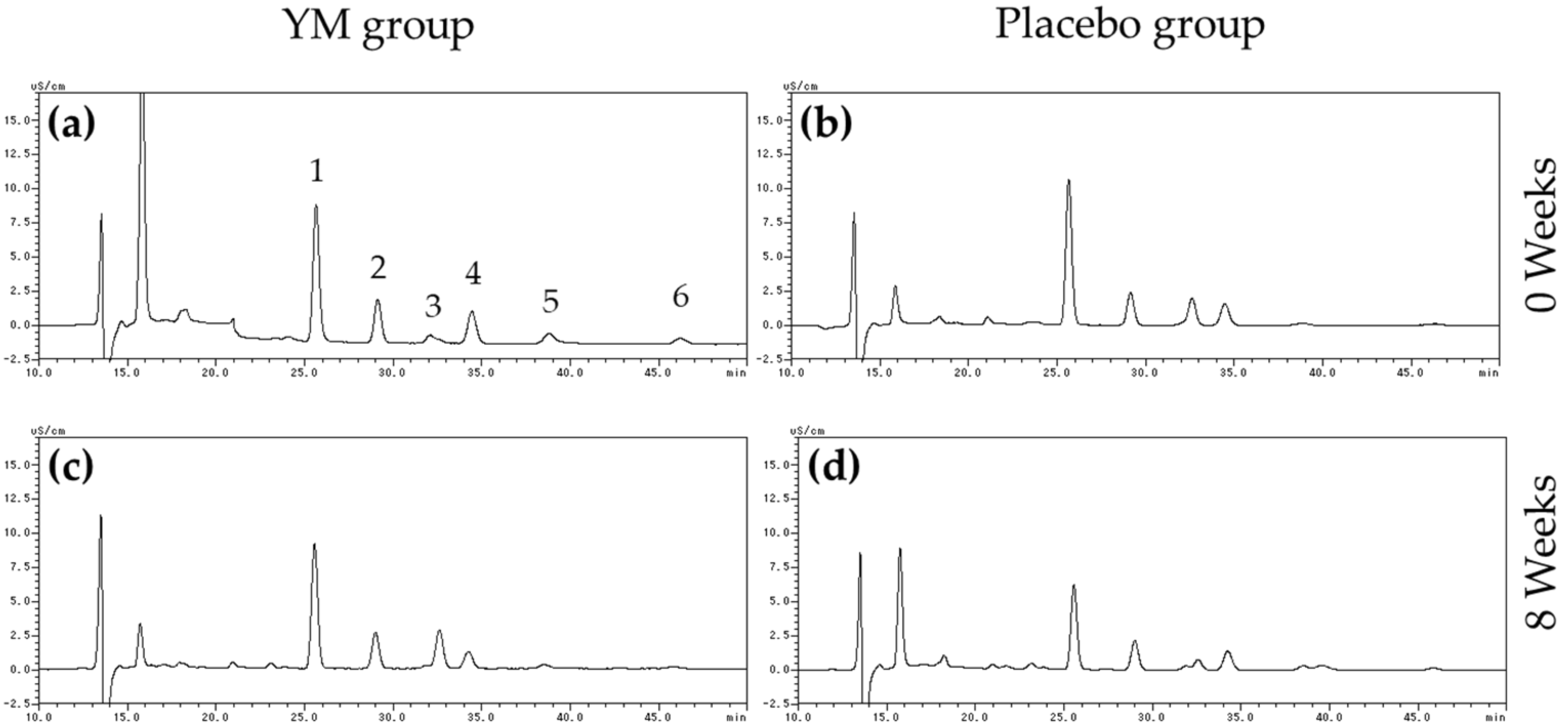

Figure S1. (Continued)

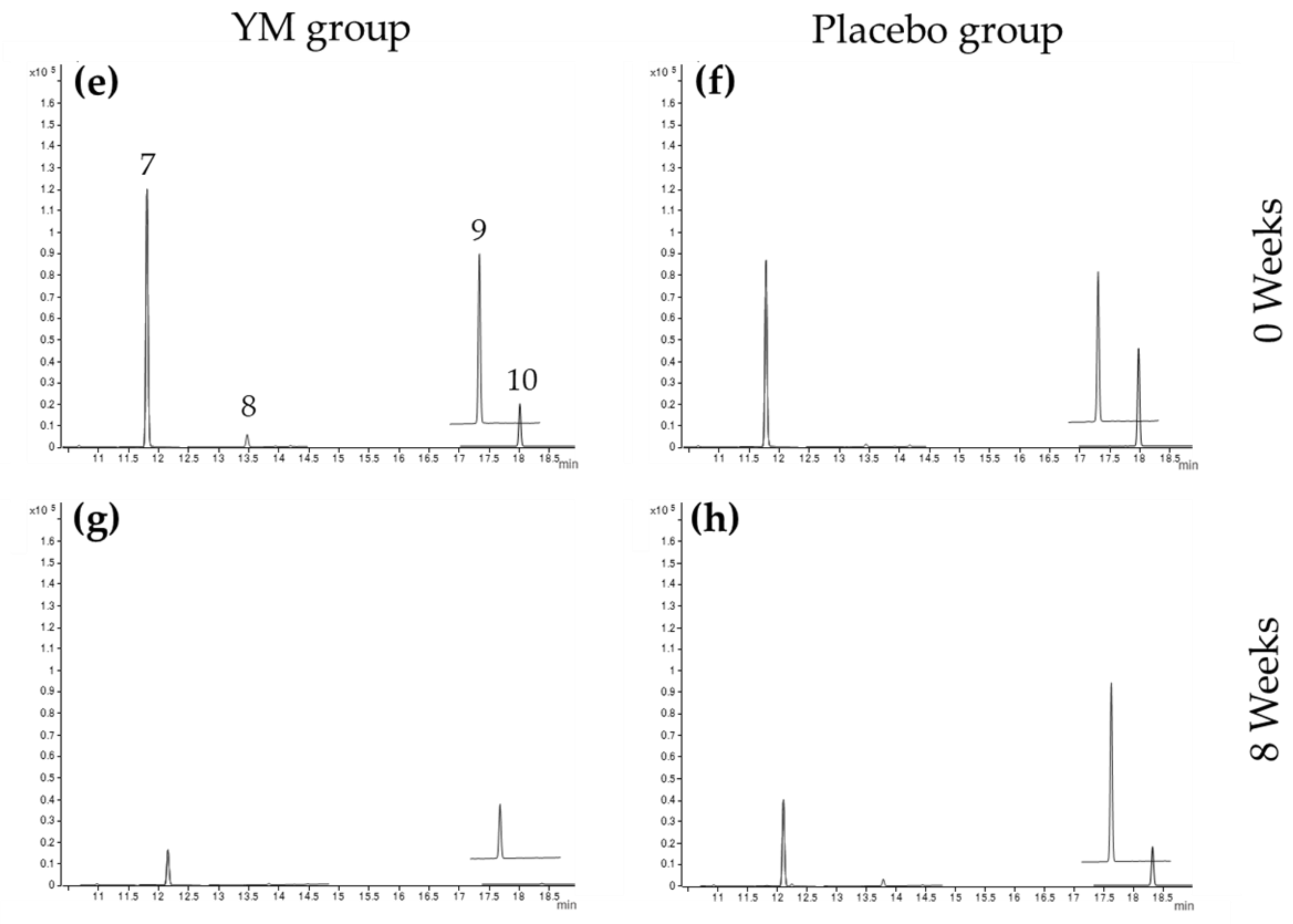

Figure S1. The typical chromatographic profiles of feces samples.

HPLC and GC-MS chromatograms in feces samples. HPLC chromatograms of YM group before intervention (a), HPLC chromatogram of Placebo group before intervention (b), HPLC chromatogram of YM group after 8 weeks intervention (c), HPLC chromatogram of Placebo group after 8 weeks intervention (d), GC-MS chromatogram of YM group before intervention (e), GC-MS chromatogram of Placebo group before intervention (f), GC-MS chromatogram of YM group after 8 weeks intervention (g), and GC-MS chromatogram of Placebo group after 8 weeks intervention (h): acetic acid (1), propionic acid (2), *iso*-butyric acid (3), *n*-butyric acid (4), *iso*-valeric acid (5), *n*-valeric acid (6), *p*-cresol (7), 4-ethylphenol (8), indole (9), skatole (10). HPLC analysis was performed using a Prominence organic acids analysis system with a CDD-10A conductivity detector (Shimadzu, Kyoto, Japan); two tandemly-arranged Shim-pack SCR-102(H) columns (300 mm × 8 mm inner diameter (i.d.)); a Shim-pack SCR-102(H) guard column (50 mm × 6 mm i.d.). 5 mM *p*-toluenesulfonic acid was used as a mobile phase and 5 mM *p*-toluenesulfonic acid with 100 μM EDTA and 20mM Bis-Tris was used as a pH buffering reagent. The flow rate was 0.8 mL/min. A column temperature was 45 °C. GC-MS analysis was performed using gas chromatography with mass spectrometry (5977A, Agilent Technologies, CA, USA) and a capillary column (DB WAX UI+G, 60 m × 0.25 mm × 0.25 μm, Agilent Technologies, CA, USA). Sample injection volume was 0.8 μl with splitless mode. Chromatography was performed under constant flow set at 1.7 ml/min. The GC oven was initially set to 70°C for 2 min after sample injection, then heated to 190°C at 25°C/min ramp rate, heated to 200°C at 2°C/min ramp rate, heated to 240°C at 25°C/min ramp rate, and kept constant at 240°C for 8 min. Mass spectra were recorded in the SIM mode. The ions monitored in SIM runs were: *m*/*z* 107.1 for *p*-cresol and 4-ethylphenol, *m*/*z* 117.1 for indole, and *m*/*z* 130.1 for skatole. SIM chromatograms were overlaid in a single GC-MS chromatogram chart. HPLC: High performance liquid chromatography. GC-MS: Gas chromatography and mass spectrometry. SIM: selective ion monitoring.
